# The Role of Intestinal Ultrasonography in Children with Inflammatory Bowel Disease

**DOI:** 10.5152/tjg.2026.25401

**Published:** 2026-01-26

**Authors:** İlksen Kökbaş, Ebru Hasbay, Betül Aksoy, Yeliz Çağan Appak, Serenay Çetinoğlu, Sinem Kahveci, Şenay Onbaşı Karabağ, Selen Güler, Maşallah Baran

**Affiliations:** 1 Department of Pediatric Gastroenterology, Hepatology and Nutrition, İzmir Katip Çelebi University Faculty of Medicine, İzmir City Hospital, İzmir, Türkiye; 2 Department of Radiology, İzmir City Hospital, İzmir, Türkiye

**Keywords:** Bowel wall thickness, inflammatory bowel disease, intestinal ultrasonography, pediatrics

## Abstract

**Background/Aims:**

Endoscopic and histopathological evaluation are important in the diagnosis and follow-up of inflammatory bowel disease (IBD), but they are invasive methods. This study aimed to evaluate the use of ultrasonography (USG), a noninvasive examination, in diagnosing and monitoring children with IBD.

**Materials and Methods:**

The study included IBD and non-IBD patients who underwent a prospective diagnostic colonoscopy. After colonoscopy, bowel wall thickness (BWT) of the patients was measured using USG. Ultrasonographic, endoscopic, histological, and laboratory findings were then compared.

**Results:**

Of the 62 patients who underwent colonoscopy, 25 (40.3%) were female. The mean age of the patients was 14.7 ± 3.5 years. Forty-three (69.4%) patients were diagnosed with ulcerative colitis, 12 (19.4%) with Crohn’s disease. Seven (11.3%) patients had normal colonoscopy findings. The BWT of the terminal ileum, left colon, and sigmoid colon were significantly higher in children with IBD. The BWT cut-off was detected as 2.55 mm in the terminal ileum, 3.35 mm in the right colon, 2.5 mm in the left colon and 2.25 mm in the sigmoid colon. When BWT in the terminal ileum and left colon increased, histopathological inflammation was significantly detected. A moderate positive correlation was observed between the C-reactive protein, erythrocyte sedimentation rate, and platelet values and the BWT values.

**Conclusion:**

Ultrasonography can be useful in the diagnosis of IBD patients. Specifically, BWT was found to be particularly sensitive and specific during the inflammatory period.

**Main Points**

- For inflammatory bowel disease patients, ultrasonography (USG), a non-invasive test, can be used to determine the activation or remission and to assess the presence of inflammation at the time of diagnosis.
- Terminal ileum intubation is possible in every pediatric patient, and USG may be helpful in evaluating the ileum in these patients.
- Fecal calprotectin is used as an inflammation marker in monitoring, but it does not provide complete information about the inflammation area. In this case, USG offers an additional advantage by identifying the inflmamation area.
- Unlike other pediatric studies, the colon and ileum were divided into segments, and the sensitivity and specificity of wall thickness measurements and cut-off values were compared with the gold standard endoscopic findings.

## Introduction

Endoscopic examinations play a significant role in the diagnosis and monitoring of the inflammatory bowel disease (IBD). However, due to the invasive nature of colonoscopy and the possibility of rare complications, caution should be taken when performing colonoscopy in children.^1^ Thus, there is a need for non-invasive alternative methods. Laboratory tests, such as fecal calprotectin and C-reactive protein (CRP), which are used especially in patient follow-up, can give an idea about the activation of the disease.^2^ These markers increase with inflammation and infection and do not indicate the localization of inflammation.^3^ Ultrasonography (USG) is an easily accessible method used in the diagnosis and follow-up of IBD in adults.^4^^-^^6^ Other imaging methods, such as magnetic resonance imaging (MRI) and computed tomography (CT), are also used in diagnosis and monitoring, but they are expensive, involve radiation, cannot be performed at the patient’s bedside, and require sedation in children.^7^ USG can detect bowel wall thickening, inflammation, and complications such as strictures or fistulae, which are key indicators of IBD activity. It can also be performed at the bedside. However, the reliability and sensitivity of USG in children with IBD remain unclear. While numerous studies exist in adults,^5^ data in pediatric patients remain limited.^6^

This study aimed to evaluate the role of intestinal USG in the diagnosis and management of IBD. In line with this objective, USG findings were compared with colonoscopy—regarded as the gold standard—as well as with laboratory and histological parameters, particularly inflammatory markers such as CRP, erythrocyte sedimentation rate (ESR), and platelet count (PLT).

## Materials And Methods

### Study Group

Patients aged 2-20 years who underwent colonoscopy in the pediatric gastroenterology clinic at Tepecik Training and Research Hospital between December 2022 and July 2024 and were either diagnosed with IBD or pre-diagnosed with IBD were included in the study.

The inclusion criteria were as follows: Patients diagnosed with IBD in the clinic and scheduled for control colonoscopy and those scheduled for diagnostic colonoscopy due to reasons such as abdominal pain, rectal bleeding, high ESR, thrombocytosis, and high CRP.

The exclusion criteria were as follows: Patients who underwent colonoscopy but had incomplete bowel preparation, with suboptimal colonoscopy, who did not consent to colonoscopy, with neurological problems, and younger than 1 year old.

After the colonoscopy procedure, all patients were evaluated with USG by the same radiologist within the same week. The patients’ ESR, CRP, and PLT values before the procedure were recorded. All patients were evaluated by comparing the ultrasonographic, colonoscopic, histopathological, and laboratory findings.

### Colonoscopy Procedure

**Colonoscopy preparation: **Patients were given a liquid diet for the last 3 days before the procedure, sennosides orally for the last 2 days, and enemas 1 day before the procedure.

**Figure 1.**
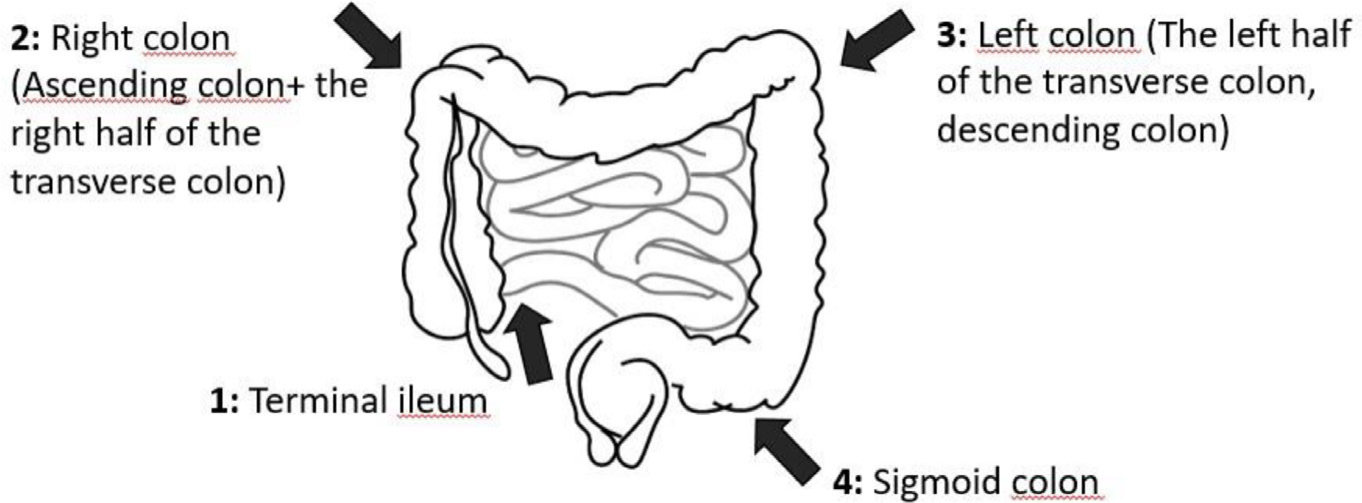
Classification of the regions examined by ultrasonography.

The procedure was performed under sedoanalgesia by the same team using a colonoscope (Fujifilm Co., Tokyo, Japan) appropriate for the patient’s age. Colonoscopy images were included in the study for comparison with the USG, which measured the terminal ileum, right colon (distal ascending–proximal transverse colon), left colon (distal transverse colon-proximal descending colon), and sigmoid colon regions (Figure 1). Inflammation was considered present in patients with ulcers, exudates, erosions, or vascular loss on endoscopy. Inflammation was considered absent if mucosal vascular and plica structures were normal.

### Histopathological Evaluation

The histopathological results of biopsies obtained from the terminal ileum, distal ascending-proximal transverse colon, distal transverse colon-proximal descending colon, and sigmoid colon were included in the study. Biopsy findings of cryptitis, crypt abscess, crypt distortion, or increased inflammatory cell infiltration were considered indicative of inflammation.^8^ Inflammation was considered present when cryptitis, crypt abscess, or crypt distortion was accompanied by inflammatory cell infiltration.

### Transabdominal Ultrasonography Procedure

Wall thickness was measured in 4 regions: terminal ileum, right colon, left colon, and sigmoid colon (Figure 1). Under the guidance of the radiologist, the terminal ileum was first identified in the right lower quadrant. In the right upper quadrant, the distal ascending colon and the proximal segment of the transverse colon were visualized. In the left upper quadrant, the distal transverse colon and the proximal descending colon were identified, while in the left lower quadrant, the sigmoid colon was assessed. From each of these four regions, at least three separate measurements were obtained, and the mean bowel wall thickness (BWT) for each region was recorded.

USG was performed according to the radiologist’s protocol, either on the same day as colonoscopy or within 1 week thereafter. In line with routine USG requirements, examinations were conducted after a 6-hour fasting period. No specific bowel filling or emptying conditions were imposed. USG was performed after colonoscopy, with the radiologist blinded to the patient’s identification number, colonoscopy results, and clinical data.

In addition to BWT, mesenteric lymphadenopathy (LAP), increased mesenteric fatty tissue, and intra-abdominal fluid were also assessed.^3^ The Philips Ultrasound iU22 device (Philips Healthcare, Bothell, WA, USA) was used with a linear probe (L12-5, 12-5 MHz).^4^

The comparison between USG and endoscopic findings was conducted by systematically recording the presence or absence of mucosal inflammation in each region defined in Figure 1, along with the corresponding BWT measurements. Likewise, for comparison with histopathological findings, BWT was documented for each region along with the presence or absence of histopathological inflammation. Furthermore, BWT measurements and additional findings were analyzed according to patient groups. All collected data were subsequently subjected to statistical analysis.

Ethical committee approval was received from theethics committee of Tepecik Training and Research Hospital (approval number: 2022/11-06; date: December 9, 2022). The patients were included in the study after receiving consent from both themselves and their parents.

### Statistical Analysis

The SPSS version 22.0 ((IBM SPSS Corp.; Armonk, NY, USA) package was used for the statistical analysis of the data, and the MedCalc 22.014 program (MedCalc Software Ltd.; Ostend, Belgium) was used for receiver operating characteristic (ROC) analysis. Compliance with a normal distribution was examined using the Kolmogorov–Smirnov test. Continuous variables that did not conform to a normal distribution were given as the median (IQR, Q1–Q3), and categorical variables were given as the number (n) and percentage (%). The Mann–Whitney *U*-test and *t*-test were used to compare 2 independent groups that did not conform to a normal distribution. Spearman correlation analysis was used to evaluate associations between continuous variables. When evaluating categorical data, the chi-square test and Fisher’s chi-square test were applied depending on suitability. *P* < .05 was considered sufficient for statistical significance.

The sample size was calculated as 62 patients to determine the performance of USG in detecting endoscopic colitis with 80% sensitivity, 80% specificity, a prevalence of 50%, a type I error of 5%, and a power of 90%.

## Results

Of the 62 patients who underwent colonoscopy, 25 (40.3%) were female. The mean age of the patients was 14.7 ± 3.5 years (median is 15.5 years). The ages of the youngest and oldest patients were 2.4 years and 20 years, respectively. Among the patients, 43 (69.4%) had ulcerative colitis (UC), 12 (19.4%) had Crohn’s disease (CD). Seven (9.7%) patients had normal colonoscopic findings. A total of 16 newly diagnosed IBD patients were included, of whom 13 had UC. The disease duration ranged from 0 to 64 months, with a mean duration of 15.8 months. The median disease duration of the patients was 10 months. In UC patients, medications included mesalamine (63%), azathioprine (56%), prednisolone (16%), and infliximab (12%); in CD patients, mesalamine (42%), azathioprine (42%), prednisolone (17%), and adalimumab (14%).

Terminal ileum intubation could not be performed in seven patients (11.3%). Two were male and 5 were female. Of these, 3 had a body weight below −2 SDS, and 1 had a body weight above +2 SDS. In 4 of these 7 patients, USG detected increased BWT in the terminal ileum, which was confirmed as terminal ileitis by MRI.

When the concordance between the colonoscopic findings and USG was evaluated, the BWT measurements of the terminal ileum, left colon, and sigmoid colon were significantly higher in patients with inflammation for all cases (*P* < .05) (Table 1). When UC, CD, and normal controls were evaluated separately, terminal ileum wall thickness was significantly increased in CD compared to both UC patients and controls. Sigmoid colon wall thickness was significantly higher in UC than in CD and normal controls, while left colon thickness was higher in UC than in CD. Mean BWT values according to groups and corresponding *P* values are presented in Table 2.

The BWT cut-off values for sensitivity and specificity varied by region in the study. Cut-off values were 2.55 mm for the terminal ileum, 3.35 mm for the right colon, 2.5 mm for the left colon, and 2.25 mm for the sigmoid colon. The regional cut-off values of the study, together with their sensitivity and specificity values, when the BWT cut-off value was set at 2 mm for the entire colon (some studies indicate this value) are shown in Table 3.

Other ultrasonographic parameters of inflammation were also evaluated. Endoscopic colitis findings were observed in 15 (65.2%) of 23/62 (37.1%) patients with mesenteric LAP. Intra-abdominal fluid was present in 8/62 (12.9%) patients and 6 of them (75%) had endoscopic colitis findings. Increased mesenteric fatty tissue was detected in 6/62 (9.7%) patients, and 5 of them (83.3%) had endoscopic colitis findings.

Regional comparison of histopathological and endoscopic findings revealed a high concordance, with histopathological inflammation frequently accompanying endoscopic inflammation (*P* < .05). At the same time, a positive correlation was observed between endoscopic and histopathological inflammation in the ileum and colon (*r* = 0.369, 0.592, respectively).

Histopathological inflammation was also compared with BWT on USG. Histopathological inflammation was detected at a high rate, consistent with increased wall thickness in the terminal ileum and left colon (Table 4). Mean terminal ileum wall thickness was 5 mm in CD and 2.74 mm in UC patients, with BWT positively correlating with histopathological inflammation. The *r* values in the terminal ileum, right colon, left colon, and sigmoid colon were determined as 0.297, 0.138, 0.329, and 0.147, respectively.

**Figure 2.**
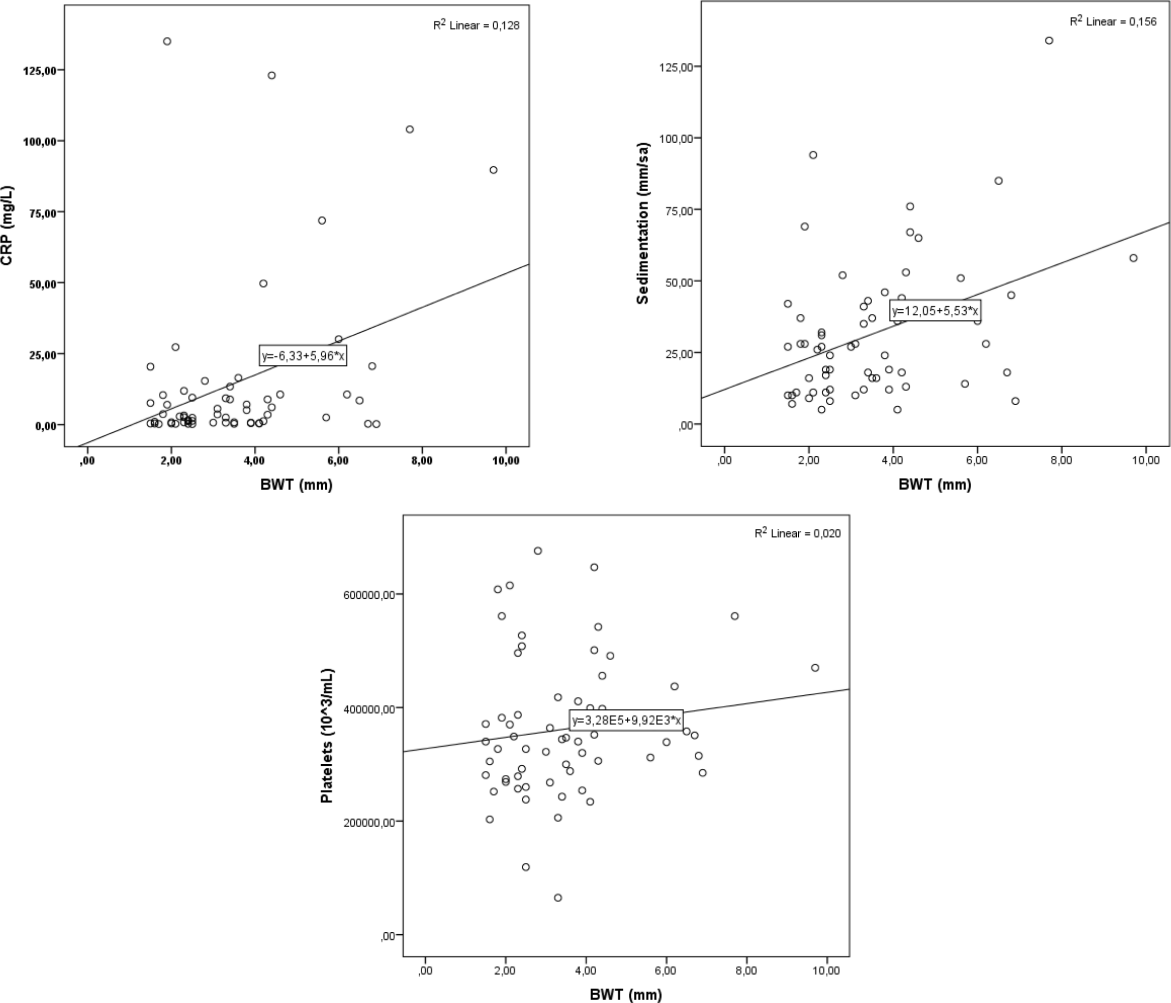
Mild correlation was detected between bowel wall thickness (BWT) and C-reactive protein (CRP), erythrocyte sedimentation rate (ESR) and platelet levels.

The BWT demonstrated a moderate positive correlation with CRP, ESR, and PLT (*r* = 0.357, 0.395, 0.173, respectively) (Figure 2). Similarly, CRP showed a moderate positive correlation with endoscopic colitis findings in the terminal ileum, right colon, left colon, and sigmoid colon (*r* = 0.282, 0.109, 0.063, and 0.083, respectively). According to BWT cut-off values, CRP and ESR were significantly higher in patients with increased wall thickness in the right and left colon compared with those without (*P* < .05). Laboratory values by other regions are summarized in Table 5. Laboratory findings were analyzed according to disease group, and patients with UC and CD showed leukocytosis, thrombocytosis, anemia, and hypoalbuminemia compared with the control group.

## Discussion

Colonoscopy is an indispensable method that allows for objective and histopathological evaluation for the diagnosis and management of IBD during exacerbations. Colonoscopy is an invasive procedure that limits its frequent use in the follow-up of pediatric patients. Although rare, colonoscopy may require special conditions and may be discontinued in cases of poor bowel preparation. In addition, ileal intubation is not possible in every patient. In this study, ileal intubation could not be performed in 7 out of 62 patients (11.3%) and in 12 out of 33 patients (36%) in another study.^9^ Therefore, alternative diagnostic and follow-up methods are gaining importance, with ultrasonographic assessment increasingly recognized in pediatric patients. In this study, the benefits of bowel wall evaluation with USG, a non-invasive and easily accessible method, in the diagnosis and during inflammation of pediatric patients with IBD were investigated.

Given the lifelong nature of IBD, radiation exposure and cost are key considerations in selecting imaging methods. USG provides real-time imaging of the intestines without exposure to radiation or the need for sedation. Additionally, it is a preferred follow-up method due to its tolerability, reproducibility, and low cost. Ultrasonography can also assess inflammation localization and extraluminal complications, including fistula, abscess, stricture, and ileus. BWT has been especially shown to be useful in the evaluation and follow-up of IBD patients in adult studies.^4^^,^^10^^,^^11^ In this study, BWT was significantly higher in the left and sigmoid colon in cases with endoscopic inflammation compared to those without (*P* ≤ .001), and a borderline difference was found in the right colon (*P* = .06). BWT cut-off values from previous studies and those obtained in the present study are presented in Table 6.^9^^12^^,^^-^^15^ According to the findings, the BWT cut-off values differed according to the regions of the colon: 3.35 mm in the right colon, 2.5 mm in the left colon and 2.25 mm in the sigmoid colon. The highest sensitivity and specificity values were observed in the left colon (84% and 78%, respectively). Sensitivity variation across intestinal regions is mainly attributable to technical aspects of USG. Terminal ileum and sigmoid colon are technically easier to assess, yielding higher sensitivity, while gas interference may reduce sensitivity in other regions. For example, a 1-cm endoscopic colonic ulcer may be missed on USG, representing a key limitation. In a study of CD patients, ileal BWT increased (mean 4.7 ± 1.9 mm) in cases with colonoscopically detected ileal inflammation.^15^ In this study, UC patients with terminal ileitis also showed a similar increase in ileal wall thickness (4.11 ± 2.25 mm). In the study, the mean age was 14.7 years vs. 11.1 years in the previous study, which may explain differences in sensitivity and specificity outcomes.

Among USG inflammation indicators, mesenteric lymph nodes have been reported as significant in adult studies,^16^^,^^17^ however; pediatric studies have not demonstrated statistically significant findings.^14^^,^^18^^,^^19^ In this study, BWT, increased mesenteric fat, and mesenteric LAP were evaluated using USG. Doppler USG and echogenicity assessment were not performed. Previous studies in adults have demonstrated a positive correlation between Limberg score, assessed by Doppler USG, and IBD activity.^20^^,^^21^ Further studies including Doppler evaluation are warranted in the pediatric population. In the study, mesenteric LAP was detected in 37.1% (23/62) of the patients, and endoscopic colitis findings were observed in 65.2% (n = 15) of these patients. Few pediatric studies have been conducted on the increase in mesenteric fatty tissue. One study reported good sensitivity and specificity (65% and 92%, respectively), whereas another found no significant findings.^9^^,^^22^ In the present study, increased mesenteric fatty tissue was detected in 9.7% (6/62) of the patients. In 83.3% (n = 5) of these cases, endoscopic colitis findings were present; however, due to the low number of patients (9.7%) with this finding, the sensitivity was considered to be low.

Histopathology provides definitive diagnostic and follow-up information but may not always align with endoscopic findings. In this study, endoscopic findings of terminal ileitis were detected in 25% (13/53) of the patients. In 92.3% (n = 12) of these patients, histopathological findings of IBD were observed. It is noteworthy that histopathological findings of IBD were observed in 62.5% (n = 25) of cases in which inflammatory findings were not detected on endoscopy. In Scomparin et al’s^23^ study, the terminal ileum was observed as normal in 72.5% (n = 58) of the patients, similar to the findings, and histopathological inflammation was detected in 51.7% (n = 30) of these patients. In the study, histopathology was normal in 7.7% of the patients with endoscopic inflammation. In another study, this rate was found to be 27.6%.^23^ In the study, a moderate correlation was observed between endoscopic and histopathological detection of ileitis and colitis (*r* = 0.369, 0.592, respectively). Similarly, Fabian et al^24^ reported a moderate correlation between microscopic inflammation severity and the Simple Endoscopic Score for CD in 63 patients.

The association between BWT and histopathological findings varied across bowel regions. In the present study, patients with histopathologically confirmed ileitis and left colonic colitis showed significantly higher BWT compared to those without inflammation (*P* = .046 and .08, respectively). However, no significant difference was found in the ultrasonographic and histopathological findings in the right colon and sigmoid colon (*P* > .05). In one study, sensitivity and specificity were found to be 68% in the terminal ileum compared with the USG and histopathological findings.^12^ In the literature, sensitivity and specificity were found to be higher in BWT in cases with histologically severe inflammatory findings (83%, 81%). If the inflammation was mild, the sensitivity of the USG was observed to decrease significantly (23%).^25^ A weak positive correlation was observed between increased intestinal wall thickness and histopathological inflammation in the ileum and entire colon (*r* = 0.138-0.329). Lower sensitivity and specificity of histopathology and USG vs. colonoscopy suggest that minimal microscopic inflammation may not substantially increase wall thickness. Studies have also shown that, as the severity of the disease increases, ultrasonographic findings become more sensitive and specific.^25^^,^^26^

In this study, BWT showed a moderate positive correlation with CRP, ESR, and PLT. There are very limited data on this subject in the literature. In an adult study by Chao Ma et al,^26^ a similar moderate correlation was found between CRP and ESR and BWT (*r* values 0.48 and 0.432, respectively). The CRP, like USG, is an easily accessible, non-invasive marker of inflammation and was evaluated alongside USG in pediatric patients. Although CRP is a useful inflammation marker, it does not show the site of intestinal involvement, limiting its utility compared with USG. Moreover, inflammation may also persist despite normal CRP levels. In the study, a moderate correlation was observed between CRP and USG findings, suggesting that their combined use may offer greater clinical utility. The CRP showed a modest positive correlation with endoscopic findings across the terminal ileum, right colon, left colon and sigmoid colon (*r* = 0.282, 0.109, 0.063, and 0.083, respectively). These findings suggest that CRP parallels endoscopic inflammation, underscoring the continued role of endoscopy and histopathology in comprehensive assessment.

Compared with healthy controls, UC and CD patients frequently showed leukocytosis, thrombocytosis, anemia, and hypoalbuminemia. These findings align with previous reports indicating that systemic inflammation and nutritional deficiencies are common in pediatric IBD.^27^ These laboratory abnormalities likely reflect active inflammation and the chronic nature of IBD. Consequently, comprehensive assessment of pediatric IBD should combine laboratory, imaging, and endoscopic findings for accurate evaluation of disease activity.

The primary aim of the study was to evaluate whether USG can serve as an indicator of inflammation in children without the need for endoscopy. As the therapeutic goal of treatment is mucosal healing, patients in remission are expected to exhibit normal endoscopic findings, whereas persistent inflammation may indicate inadequate treatment response. Therefore, it is not believed that the medications used in these patients would have influenced the outcomes relevant to the objectives of the study.

Radiological imaging studies in IBD have primarily utilized MRI and CT. The MR enterography demonstrated a sensitivity of 57% and specificity of 75%-100% for IBD diagnosis.^13^ Contrast-enhanced MRI demonstrated 84% sensitivity and 100% specificity for the terminal ileum.^28^ CT demonstrates low sensitivity (68%) for detecting bowel inflammation.^29^ MRI is an expensive method with a long scanning time and a need for sedation in young children. CT involves radiation exposure, and neither CT nor MRI can be performed at the bedside. Despite the advantages of USG, it also has disadvantages. Measurement outcomes may vary according to the examiner’s experience. Patient compliance is crucial, and peristalsis-related variations in haustra and folds should be considered during measurement. To ensure consistency and reduce inter-observer variability, all measurements were performed by a single experienced radiologist using the same device in the present study.

Considering the weight and height values that vary with age in pediatric patients, unlike in adults, it is thought that this may also affect the intestinal wall thickness.^17^ This situation may be a problem for standardization. In terms of standardization, it is necessary to study a larger number of cases and create data according to regions. Increased intestinal wall thickness is a non-specific finding and can also be seen with infectious, ischemic, and other inflammatory causes. What is detected here is the visualization of the inflammatory response of the intestine.^12^ Therefore, colonoscopic and histopathological examinations maintain their importance in the diagnosis of IBD.

Transabdominal USG is used to measure intestinal wall thickness in adults and to identify IBD complications. It is increasingly used in gastroenterology practice because it is an easily accessible and non-invasive method. However, there are different data regarding sensitivity, specificity, and accuracy in children. Studies have shown that USG can be useful in the follow-up of IBD patients and can raise awareness in the diagnosis of patients. In the present study, it was observed that among the parameters measured by USG, BWT was especially sensitive and specific in IBD patients. It would be beneficial to conduct studies with larger sample sizes to standardize BWT cut-off values in children.

## Figures and Tables

**Table 1.** Ultrasonographic Bowel Wall Thickness According to Colonoscopic Inflammation

	**Inflammatory Findings at Colonoscopy**		
**The Area Evaluated in USG** **(Bowel Wall Thickness)**	**Present**	**Absent**	*P* **^+^**
Terminal ileum (mean ± SD) (mm)	4.11 ± 2.25	2.50 ± 0.73	**.007**
Right colon (mean ± SD) (mm)	3.12 ± 1.53	2.41 ± 0.76	.061
Left colon (mean ± SD) (mm)	4.04 ± 1.78	2.38 ± 1.18	**<.001**
Sigmoid colon (mean ± SD) (mm)	3.57 ± 1.74	2.27 ± 1.10	**.001**

*P* < .05 was considered statistically significant

^+^Mann–Whitney *U*-test.

mm, millimeter; SD, standard deviation.

**Table 2.** Ultrasonographic Bowel Wall Thickness Across Disease Groups by Intestinal Segment

**Intestinal Segment**	**Normal**	**Ulcerative Colitis**	**Crohn Diseases**	*P* *
Terminal ileum	^1^2.37 ± 0.5	^2^2.6 ± 0.8	^3^4.36 ± 2.28	*P*^1^^-2^ = .472 *P*^1^^-3^ = **.038** *P*^2^^-3^ = **.000**
Left colon	^1^2.43 ± 0.85	^2^3.49 ± 1.81	^3^2 ± 0.57	*P*^1^^-2^ = .139 *P*^1^^-3^ = .204 *P*^2^^-3^ = **.007**
Right colon	^1^2.41 ± 0.43	^2^2.75 ± 1.22	^3^2.48 ± 1.08	*P*^1^^-2^ = .483 *P*^1^^-3^ = .874 *P*^2^^-3^ = .503
Sigmoid colon	^1^2.16 ± 0.31	^2^3.4 ± 1.77	^3^2 ± 0.65	*P*^1^^-2^ = **.073** *P*^1^^-3^ = .578 *P*^2^^-3^ = **.011**

*P* < .05 was considered statistically significant.

*Independent sample *t*-test.

Values represent ultrasonographic bowel wall thickness (mm), expressed as mean ± SD.

**Table 3.** Assesment of Bowel Wall Thickness (mm) by Transabdominal Ultrasonography (USG) at Different Intestinal Sites According to Cut-Off Value

**Region**	**AUC**	**95% CI (Lower)**	**95% CI (Upper)**	*P*	**Cut-off (mm)**	**Sensitivity (%)**	**Specificity (%)**	**PPV**	**NPV**
Terminal ileum	0.589	0.377	0.713	.779	>2.55	75	63	97.5	11.8
Right colon	0.710	0.551	0.838	**.008**	>3.35	40	96	99.4	7.7
Left colon	0.792	0.673	0.911	**<.001**	>2.5	84	78	98.6	20.4
Sigmoid colon	0.769	0.641	0.897	.001	>2.25	83	74	96.6	42.8
Colon	0.669	0.538	0.784	**.015**	>2	84	28	74	41.7

*P* < .05 was considered statistically significant. Receiver Operating Characteristic (ROC) analysis.

AUC, area under curve; mm, millimeter; NPV, negative predictive value; PPV, positive predictive value.

**Table 4.** Comparison of Ultrasonographic Bowel Wall Thickness and Histopathological Findings

**Intestinal Segment**	**Histopathologic Inflammation**		
	**Present**	**Absent**	*P* **^+^**
Terminal ileum	3.19 ± 1.61	2.29 ± 0.50	**.046**
Right colon	2.70 ± 1.01	2.59 ± 1.31	.31
Left colon	3.51 ± 1.82	2.22 ± 0.84	**.008**
Sigmoid colon	3.40 ± 1.82	2.67 ± 1.40	.12

*P* < .05 was considered statistically significant.

^+^ Mann–Whitney *U*-test.

Values represent ultrasonographic bowel wall thickness (mm), expressed as mean ± SD.

**Table 5.** Comparison Between C-reactive Protein (CRP), Erythrocyte Sedimentation Rate (ESR) and Platelet Values According to Bowel Wall Thickness (BWT) Cut-Off Values

**BWT Cut-Off Values (mm)**	**CRP (mg/L)** **(Mean ± SD)**	**ESR (mm/sa)** **(Mean ± SD)**	**Platelet (10^3^/μL)** **(Mean ± SD)**
Terminal ileum ≥2.55 <2.55 *P*	22.1 ± 33.7 8.6 ± 23.3 .18	41.1 ± 27.8 23.6 ± 18 .15	399 ± 112 332 ± 124 .92
Right colon ≥3.35 <3.35 *P*	33 ± 44.9 10.3 ± 22.3 **<.001**	51.2 ± 32.9 26.9 ± 19.5 .08	413 ± 99 350 ± 125 .35
Left colon ≥2.5 <2.5 *P*	22.0 ± 33.2 8.3 ± 23.6 **.02**	39 ± 28.4 25.0 ± 18.4 **.03**	383 ± 118 344 ± 125 .89
Sigmoid colon ≥2.25 <2.25 *P*	19.2 ± 30.3 10.8 ± 28.1 .43	37.3 ± 27.6 25.1 ± 20.4 .28	372 ± 119 349 ± 141 .38

BWT, bowel wall thickness; CRP, C-reactive protein; ESR, erythrocyte sedimentation rate; mm, millimeter; SD, standard deviation.

Mann–Whitney *U*-test.

**Table 6.** Current Study and Other Studies in the Literature That Correlated Colonoscopy with Ultrasonography

	Mean/Median*Age	**Number of Patients**	**Cut-off (mm)**	**Sensitivity (%)**	**Specificity (%)**
This study	14.7 ± 3.5 15.5*	62	TI 2.55 RC 3.35 LC 2.5 SC 2.05	TI 75 AC+TC 40 DC+TC 84 SC 87	63 96 78 58
Bremner^9^	12*	44	TI 2.5 C 3	TI 75 AC 46 TC 67 DC 54 SC 50	92 88 90 100 88
Haber^12^	11.2	78	TI 1.5 C 2	TI 100 AC 72 TC 74 DC 74	72 81 94 89
Ziech^13^	14	28	3	55	100
Civitelli^14^		50	3	RC 75 TC 86 LC 96	100 100 100
Faure^15^	11.1	38	TI 2.5 C 2	TI 100 RC 77 TC 80 SC 93	92 91 90 100

AC, ascending colon; C, colon; DC. descending colon; LC, left colon; mm, millimeter; RC, right colon; SC, sigmoid colon; TC, transvers colon; TI, terminal ileum.

## Data Availability

The data that support the findings of this study are available on request from the corresponding author.
